# Behavioral, Medical Imaging and Histopathological Features of a New Rat Model of Bone Cancer Pain

**DOI:** 10.1371/journal.pone.0013774

**Published:** 2010-10-29

**Authors:** Louis Doré-Savard, Valérie Otis, Karine Belleville, Myriam Lemire, Mélanie Archambault, Luc Tremblay, Jean-François Beaudoin, Nicolas Beaudet, Roger Lecomte, Martin Lepage, Louis Gendron, Philippe Sarret

**Affiliations:** 1 Department of Physiology and Biophysics, Université de Sherbrooke, Sherbrooke, Quebec, Canada; 2 Department of Nuclear Medicine and Radiobiology and Centre d'Imagerie Moléculaire de Sherbrooke, Université de Sherbrooke, Sherbrooke, Quebec, Canada; Cedars-Sinai Medical Center and University of California Los Angeles, United States of America

## Abstract

Pre-clinical bone cancer pain models mimicking the human condition are required to respond to clinical realities. Breast or prostate cancer patients coping with bone metastases experience intractable pain, which affects their quality of life. Advanced monitoring is thus required to clarify bone cancer pain mechanisms and refine treatments. In our model of rat femoral mammary carcinoma MRMT-1 cell implantation, pain onset and tumor growth were monitored for 21 days. The surgical procedure performed without arthrotomy allowed recording of incidental pain in free-moving rats. Along with the gradual development of mechanical allodynia and hyperalgesia, behavioral signs of ambulatory pain were detected at day 14 by using a dynamic weight-bearing apparatus. Osteopenia was revealed from day 14 concomitantly with disorganization of the trabecular architecture (µCT). Bone metastases were visualized as early as day 8 by MRI (T_1_-Gd-DTPA) before pain detection. PET (Na^18^F) co-registration revealed intra-osseous activity, as determined by anatomical superimposition over MRI in accordance with osteoclastic hyperactivity (TRAP staining). Pain and bone destruction were aggravated with time. Bone remodeling was accompanied by c-Fos (spinal) and ATF3 (DRG) neuronal activation, sustained by astrocyte (GFAP) and microglia (Iba1) reactivity in lumbar spinal cord. Our animal model demonstrates the importance of simultaneously recording pain and tumor progression and will allow us to better characterize therapeutic strategies in the future.

## Introduction

Among cancers with a poor prognosis, those originating from breast, lung and prostate commonly metastasize to the skeleton [1], [2]. While primary bone cancers are rare, over 70% of people coping with advanced breast or prostate cancer will develop bone metastases [3]. The major factor responsible for the decrease in quality of life in bone metastases-bearing patients is pain [4].

Bone cancer pain originates from nerve compression, ischemia and release of proinflammatory substances by the tumor and cells from the bone environment [5]. Thus, lytic cancer cells affect bone homeostasis through the release of cytokines promoting pathological resorption by osteoclasts [6], [7]. In addition to bone destruction [8], osteoclast hyperactivity creates an acidic environment responsible for the accommodation of pain [9]. Compensative and anarchic bone formation by osteoblasts compresses free nerve terminal endings scattered in the bone marrow, matrix and periosteum [10], leading to the genesis and maintenance of pain. The association of those phenomena produces a unique mechanical and neurochemical onset that goes beyond the combination of neuropathic and inflammatory pain [11].

Bone cancer pain's unique characteristics make it therapeutically intractable [11]. Indeed, typical approaches including anti-inflammatory drugs and opiates have limited relief efficiency [12]. Due to its progressive nature, escalating doses are required to adequately alleviate bone cancer pain [13], leading to side effects and ultimately to abortive compliance [4], [14]. Moreover, morphine was recently proven to accelerate cancer-induced bone loss in osteolytic cancers [15], [16]. Similarly, promising anti-resorptive bisphosphonate therapies [17], [18], [19], [20], [21], shown to slow cancer progression and indirectly alleviate pain, were shaded by comorbid osteonecrosis [22]. Bone cancer pain thus remains a concern, highlighting the need to accentuate our understanding of its neurobiological mechanisms in order to develop more effective strategies.

Animal models were elaborated to mimic the human condition in an attempt to understand bone cancer pain. Mouse models of calcaneus and humerus cancer [23], or rat models of tibia cancer with mammary MRMT-1 [24], 4T.1 [25] and Walker 256 [26], or prostatic AT-3.1 [27] cells were developed to characterize local changes or neural plasticity of spinal structures projecting to the bone. From a clinical point of view, the femur, however, represents the most frequently affected long bone [28]. Mouse femoral models involving intramedullary implantation of fibroblast cancer cell lines to induce pain are reported (e.g. fibrosarcoma [29], [30]). However, those models are based on mimicking primary bone cancers. The majority of secondary bone cancers originate from breast or prostate cancers, known for their bone-metastasizing properties [31]. Yet, for models involving those types of cells, induction is either intravenous (leading to multiple site metastases [32], [33]) or follows a surgical procedure in which a patellar arthrotomia and partial alteration of the knee joint are susceptible to impaired locomotion, which in turn biases the evaluation of pain-related behavior. Indeed, invasiveness and surgical procedures need to remain minimal in order to assess breakthrough pain without interference. This latter pain manifestation is the most refractory condition to overcome for individuals coping with chronic cancer pain and among the toughest to evaluate in animal models.

In addition to pain assessment, the means to detect and monitor bone pathologies have evolved to incorporate X-ray micro-computed tomodensitometry (µCT) and bone scintigraphy [34]. Nonetheless, preventive determination of tumor nesting and progression has the potential to improve analgesic posologies and to limit bone remodeling. Advanced non-invasive and low radiation medical imaging, such as positron emission tomography (PET) and magnetic resonance imaging (MRI) or the combination of both, would be desirable to enable efficient and repeated exams for accurate and rigorous analgesic therapy adjustments [35].

The rat remains the most studied species in pain paradigms [36]. We therefore selected a rat mammary carcinoma cell line for its spontaneously metastasizing and transplantable properties. The capability of MRMT-1 to metastasize to remote organs is intrinsic to those syngeneic cells [37]. In the present study, we describe an intervention in the Sprague-Dawley rat femur to implant breast tumor cells using a light surgical procedure in an effort to mimic clinical pain. We utilized a combination of innovative behavioral and medical imaging approaches, supported by immunohistochemical and pathological observations, to characterize the model. The study aims at improving clinical relevance with regard to the model and tools used to screen new lead compounds, in order to characterize bone tumor progression as well as to elucidate the mechanisms underlying the genesis and maintenance of bone cancer pain.

## Methods

### Cell culture

Mammary rat metastasis tumor (MRMT-1) cells (carcinoma) were kindly provided by the Cell Resource Center for Biomedical Research Institute of Development, Aging and Cancer (Tohoku University 4-1, Seiryo, Aoba-ku, Sendai, Japan) and harvested in RPMI 1640 medium (Gibco, Montreal, Quebec, Canada) supplemented with 10% fetal bovine serum (heat-inactivated) and 2% penicillin/streptavidin (Gibco, Montreal, Qc, Canada). Cells were detached by brief exposure to 0.25% w/v trypsin-EDTA (Gibco, Montreal, Qc, Canada) and prepared for injections. Briefly, cells were pelleted by centrifugation (3 min. at 200×g), rinsed with 1 ml of Hank's balanced salt solution exempt of calcium, magnesium or phenol (HBSS; Gibco, Montreal, Qc, Canada) and further centrifuged in the same conditions. The pellet was re-suspended in 1 ml of HBSS, and cells were counted with a hemocytometer. Cells were diluted to achieve a final concentration for injection of 30000 cells in 20 µl and maintained on ice prior to surgery.

### Animals

Adult male Sprague-Dawley rats (200–225 g; Charles River Laboratories, St.-Constant, Quebec, Canada) were maintained on a 12 h light/dark cycle with access to food and water *ad libitum*. Animal-related procedures were approved by the Ethical Committee for Animal Care and Experimentation of the Université de Sherbrooke and carried out according to the regulations of the Canadian Council on Animal Care (CCAC). Rats were acclimatized for 4 days to the animal facility and for 2 days to manipulations and devices prior to behavioral studies. Note that animals tested for pain and used for immunohistochemical analyses were different from those being imaged by MRI or MRI-PET, since prolonged anesthesia can affect behavioral responses.

### Surgery

After complete anesthesia with 5% isoflurane (Abbott Laboratories, Montreal, Qc, Canada), rats were laid in the supine position and their right paws were shaved and disinfected with 70% v/v ethanol. Anesthesia was maintained with 2.5% isoflurane and the rat eyes were protected with an ophthalmic liquid gel (Tear-gel, Novartis, Mississauga, ON). A minimal skin incision (8–10 mm) exposed the *quadriceps femoris*. The *vastus lateralis* was incised (5–8 mm in length) to expose the femoral epicondyle, while the patellar ligament remained untouched and minimal damage was inflicted to the surrounding muscle and blood vessels. Using a 0.8 A stereotaxic drill (Foredom, Bethel, CT, USA) connected to a 1.75 mm carbide steel burr (Stoelting Co., WoodDale, IL, USA), a small and superficial cavity was burred between the medial epicondyle and the adductor tubercle (approximately 1 mm in depth). In that cavity, a 25-gauge needle was inserted at a 45° angle, allowing it to reach the intramedullary canal of the femur. The needle was substituted with a blunt-end 25-gauge needle connected to a 50 µl Hamilton syringe containing 20 µl of the cell suspension (cancer group) or of HBSS (sham group). The syringe was left in place for 1 min to allow cell dispersion in the bone marrow. The needle was then removed and the cavity was sealed with dental amalgam (Prodigy A3, Kerr, Orange, CA) and polymerized with a curing light (QHL75, Dentsply, Milford, DE). The site was thoroughly washed with sterile deionized water. The muscle and the conjunctive tissue were closed with a discontinuous suture made with synthetic absorbable sutures (Monocryl, Ethicon, Sommerville, NJ), and the skin was closed with a continuous suture from non-absorbable surgical suture (Prolene, Ethicon, Sommerville, NJ). The wound was finally washed with 3% v/v peroxide and sprayed with a bitter solution (Aventix Animal health, Flamborough, ON).

### Behavioral studies

*Mechanical allodynia* was assessed using a dynamic plantar esthesiometer (Ugo Basile, Stoelting, IL, USA), an automated version of the von Frey hair. Animals were placed in enclosures on an elevated wire mesh and responses to punctate mechanical stimulation were assessed using the esthesiometer. A straight metal filament (0.5 mm diameter) was orientated beneath the pad until it touched the plantar surface of the hind paw and began to exert an upward force. The force required to elicit a withdrawal response was measured in grams and automatically registered when the paw was withdrawn or the preset cut-off was reached (50 g). Five values were taken alternately on both ipsilateral (operated side) and contralateral hind paws at intervals of 15 s.

*Mechanical nociceptive withdrawal responses* were measured using an analgesiameter (Ugo Basile, Stoelting, IL, USA) that applies a linearly increasing pressure (16 g/s) via an acrylic stylus. The stylus was placed over the medial dorsum of the hind paw, between the 1st and 2nd tarsometatarsal joints and the mean of four consecutive measures beginning with the left or right hind paw, alternately, was recorded as the withdrawal threshold in grams. The cut-off was set to 200 g. Comparison for each testing day was performed by subtracting the ipsilateral weight (g) from the contralateral weight value (g) to determine the paw withdrawal threshold (PWT) difference.

*Static weight bearing* was measured using the Incapacitance Meter (IITC Life Science, Woodland Hills, CA, USA). The animal was positioned with the hind limbs at rest on the two weight-averaging platform pads. The unit recorded the average weight put on each pad over a 2 s period. The measurement was repeated 3 times. The results obtained with the incapacitance meter were used as a control for a second weight-bearing test, the Dynamic Weight Bearing test (Bioseb, Boulogne, France). The device consisted of a bottomless Plexiglas enclosure (25×25×24 cm^3^) and a sensor (1936 single captors). The sensor was placed on the floor of the enclosure, covering its entire surface. The rat was allowed to move freely within the apparatus for 5 min and the weight-bearing information was transmitted live to a laptop computer via an USB interface. The raw data were analyzed with DWB 1.1.0.6 software (Bioseb). A paw was detected when one captor recorded a weight of at least 4 g and a minimum of 2 adjacent captors recorded a weight of at least 1 g. The paw had to be stable for a minimum of 0.5 s to be included. Using a synchronized video-recording of the test and the scaled map of the detected zones, each presumed paw was validated by an observer and identified as a left or right and fore or hind paw. Other zones detected, representing the tail or another body part, were also included in the analysis.

*The effect of subcutaneous morphine* was evaluated on days 11, 14, 18 and 21 in our model. Morphine-sulphate stock solution (50 mg/ml) was diluted in sterile saline to achieve injection concentration (3 mg/ml). Rats were than injected s.c. with 3 mg/kg (1 µl/g) and returned to their cage. Behavioral tests were performed 30 minutes after the injection as described earlier.

### Immunohistochemistry

Rats were deeply anaesthetized with 5% isoflurane and transcardially perfused with 4% paraformaldehyde (PFA) in phosphate buffer (0.1 M, pH 7.4). The spinal cord and dorsal root ganglia (DRG) were post-fixed for 30 min in 4% paraformaldehyde and then transferred to 30% sucrose (48 h) for cryoprotection. Serial frozen spinal cord sections, 35 µm thick, were cut using a sliding microtome, collected in PBS and processed as free-floating sections. Frozen DRG were embedded in OCT Tissue-Tek, cut on a cryostat at a thickness of 12 µm, collected and processed on slides.

Tissue sections were incubated for 60 min at room temperature in a blocking solution of 5% normal goat serum in PBS with 0.5% Triton X-100 and 2% albumin and then incubated for 30 min in 0.1 M glycine solution to reduce autofluorescence. The sections were then incubated with the primary antiserum overnight at 4°C. Spinal cord sections were immunostained with antibodies against markers of microglia, ionized calcium binding adaptor molecule 1 (Iba1, polyclonal antibody rabbit anti-Iba1, 1∶1000, Wako, Richmond, VA, USA), astrocytes, glial fibrillary acidic protein (GFAP, monoclonal clone GA5 mouse anti-GFAP, 1∶500, Sigma, Oakville, Ontario, Canada) and c-Fos protein (c-Fos, polyclonal rabbit anti-Fos, 1∶20 000, Santa Cruz Biotechnology Inc., Santa Cruz, CA, USA) and in DRG against activating transcription factor 3 (ATF-3 (C-19), polyclonal rabbit anti-ATF-3, 1∶500, Santa Cruz Biotechnology Inc., Santa Cruz, CA, USA). After incubation, tissue sections were washed two times for 10 min in PBS and incubated in the secondary antibody solution for 2 h at room temperature. Secondary antibodies conjugated to fluorescent markers Alexa Fluor® 488 and Alexa Fluor 647® were used at 1∶500 (Invitrogen Molecular Probes, Eugene, OR, USA). Sections were then mounted with Aquamount. The absence of cross-reactivity among the secondary antibodies was verified by omitting primary antibody during the overnight incubation.

Fos protein and ATF-3 factor were also detected by immunohistochemistry, using the avidin-biotin-peroxidase method. The DRG slides and spinal cord sections were treated with 0.5% hydrogen peroxide for 30 min to inhibit endogenous peroxidase activity and incubated for 30 min in a blocking solution of 5% normal goat serum, 2% albumin, and 0.5% Triton X-100 in PBS. The sections were then incubated overnight at 4°C in the primary c-Fos (spinal cord) and ATF-3 (DRG) antibodies, which were diluted in 1% NGS with 0.1% Triton X-100. After incubation, the sections were rinsed and blocked with 3% NGS for 15 min and incubated for 2 h in the secondary antibody, biotinylated goat anti-rabbit IgG (goat anti-rabbit H+L, 1∶500, Vector Laboratories, Burlingame, CA, USA), followed by streptavidin-horseradish peroxidase with the avidin-biotin method for 1 h (Horseradish Peroxidase Streptavidin, PK-6100, Vector Laboratories, Burlingame, CA, USA) at room temperature. Sections were then incubated for 3 min with 0.02% diaminobenzidine with 0.01% hydrogen peroxide, then dehydrated and coverslipped with Permount® Mounting Media (SP15-500, Fisher Scientific, NY, USA).

### Quantification

Using an Olympus Confocal Imaging microscope and Olympus Fluoview FV1000 software, sections from the lumbar spinal cord were taken with identical acquisition parameters (gain, exposure time) and analyzed by confocal microscopy to characterize immunofluorescence (IF). Epifluorescence and bright-field pictures were taken by a Leica DM4000 microscope equipped with a Leica DFC350FX and an InfinityX camera for immunofluorescence.

Fos-immunoreactive nuclei were counted when having small, rounded or ovoid, dark staining, in comparison to control. Laminae delimitation was performed with a rat spinal cord histo-architecture template [38], in accordance with lumbar segment L1/L2/L3 of the section. In the present study, c-Fos-immunoreactive cells were counted as combined laminae I-II, III-IV, V-VI, and VII-VIII, respectively. Eight 35-µm spinal cord sections over the L1/L2/L3 segment of each animal (n = 3) were randomly chosen per animal per condition to determine total c-Fos number.

The quantification of ATF-3 positive neurons in DRG 12-µm-thick sections was carried out by counting the number of neurons with ATF-3 immunoreactive nuclei at 20X magnification, using a Leica DFC350FX microscope. DRGs corresponding to the lumbar spinal cord levels L1/L2/L3 were selected as the main projection sites of primary afferent fibers innervating the femur [39], [40]. Counting of ATF-3 positive cells was performed once in three series, every 36 µm. This count was expressed as mean number of ATF-3 immunoreactive cells per section (n = 24). Sham and cancer animals from 3 independent experiments were used for ATF-3 determination.

Glial staining for GFAP and Iba-1 in the L1/L2/L3 spinal cord was quantitatively analyzed by optical density with Image J software version 1.6. Image J was first calibrated with an image from a control animal using the calibrate function. The settings were then conserved for all subsequent analyses. Three randomly selected sections of the spinal cord L1-L3 in each animal (n = 3) were analyzed. The optical densities of nine sections (three sections/animal) were averaged to provide a mean number for each condition.

### Radiological analysis

Radiographs were performed on dissected bones using a Faxitron MX-20 (Faxitron, Lincolnshire, IL). Pictures were taken at 26 kV, exposure time: 10 s; magnification: 2X. Micro-computed tomography analyses were performed on dissected limbs of tested rats with a 1072 MicroCT system (Skyscan, Kontich, Belgium). The parameters were: source: 80 kV, 124 µA; zoom: 20X; pixel size: 14.06 µm; exposure time: 3.0 s; rotation: 0.9°. Bone Volume/Total Volume (BV/TV) determination: cortical + trabecular bone volume (BV) divided by any type of tissue (TV) in the range of 5,626 mm corresponding to 201 cross sections (volume of interest; VOI), starting from the growth plate of the distal femur. Trabecular BV/TV is defined by the trabecular bone volume divided by the TV from the VOI. Analyses were performed with CT Analyser 1.10.0.1 (Skyscan, Kontich, Belgium).

### Magnetic Resonance Imaging and Positron Emission Tomography

MRI studies were conducted at the *Centre d'imagerie moléculaire de Sherbrooke* with a 210 mm small-animal 7T scanner (Varian Inc., Palo Alto, CA, USA) and a 63 mm volume RF coil. Sprague-Dawley rats were placed in the supine position in an MRI-compatible cradle equipped with a homebuilt paw support designed to position both limbs stably and reproducibly. Animals were anaesthetized with 3% (induction) and 1.5% (stabilization) isoflurane in oxygen. A feedback-controlled animal warm-air heater system was used to keep the animal's body temperature at physiological levels and the respiration rate was continuously monitored (SA Instruments Inc., Stony Brook, NY, USA). The MRI protocol included the acquisition of axial (sagittal) pre-contrast and 10 min post-contrast *T*_1_-weighted images, using a gradient echo sequence with TR: 210 ms, TE: 3.35 ms, flip angle: 30°, matrix: 256×256, FOV: 60×60 mm^2^, NA: 8 (30 sagittal slices), 1.5 mm thick. A 600-µl bolus of contrast agent (Gd-DPTA, Magnevist, Berlex) was injected via the tail vein. Animals were imaged before and 6, 8, 10, 13, 15 and 18 days post-implantation. To evaluate tumor invasiveness, a region of interest (ROI), including the whole bone cortex and medullar channel of the femur, was defined. This ROI yielded a voxel intensity histogram for both hind paws. The contralateral maximal voxel intensities were used to fix the pathological threshold. Every ipsilateral voxel remaining was colored according to its intensity with a yellow-red scale. Mathlab Version 7.1 (Mathworks Inc., Natick, Massachusetts, USA) was used to analyze the data and perform the calculations.

Positron emission tomography was performed using the LabPET4 Sherbrooke with Core Temperature Control. Ring diameter: 162 mm; FOV: 37.5 mm; Cristals: 3072; Spatial resolution: 1.35 mm FWHM FOV; Noise-equivalent counts: 37 kcps at 245 MBq (250–650 keV). Immediately after MRI scans, animals remained under anesthesia in the same position and the cradle was transferred to the PET scanner. The collimator was aligned to the hind knee joints; 9.25 MBq of Na^18^F was injected i.v. (200 µl at 500 µl/min). The distribution of the radiotracer was monitored over 30 min and double-volume sampling was performed for 30 min. Breathing and body temperature of the animal were monitored at all times throughout the procedure.

### Histological analysis and measurement of osteoclast activity

Twenty-one days after tumor injection, rats were euthanized and femora were removed. Femurs were fixed in 4% paraformaldehyde for 72 hours, decalcified in 10% EDTA (pH 7.4) for 2–3 weeks, and finally embedded in paraffin. Paraffin blocks were cut in 3- or 5 µm-thick sections with a microtome equipped with a carbide blade and slices were stained with Harris' hematoxylin and eosin to determine cancer cell infiltration and bone destruction. To identify activated osteoclasts, tartrate-resistant acid phosphate (TRAP) staining was performed. Using the Leukocyte (TRAP) Kit (Sigma-Aldrich, St. Louis, Missouri, United States), the slices were exposed to the substrate for 1 hour and staining was visualized with a Leica DM4000B (Leica, Toronto, ON, Canada). In total, 3.5 fields were counted at a magnification of 250X for a total surface of 1 mm^2^. Labeled osteoclasts were counted along the mineralized bone-tumor or the bone-bone marrow interfaces and 4–7 slices were analyzed for each animal, from groups of 4 animals.

### Statistical analyses

Von Frey and DWB data were analyzed using a two-way ANOVA followed by Bonferroni's post-hoc test and Randall-Sellitto was analyzed with a Student's *t*-test for comparison of contra- and ipsilateral hind paws for each day. C-Fos, ATF-3 and Iba1/GFAP count differences were determined with a repeated measure ANOVA followed by a Bonferroni multiple-comparison test. Normality was evaluated with Kolmogorov-Smirnov and Shapiro-Wilks tests. BV/TV graphs were analyzed with a one-way ANOVA and TRAP counts with a Student's t-test. P≤0.05 was considered as the level of statistical significance.

## Results

### Implantation of breast carcinoma cells in the femur induces touch-evoked and ambulatory pain

**Figure 1:**
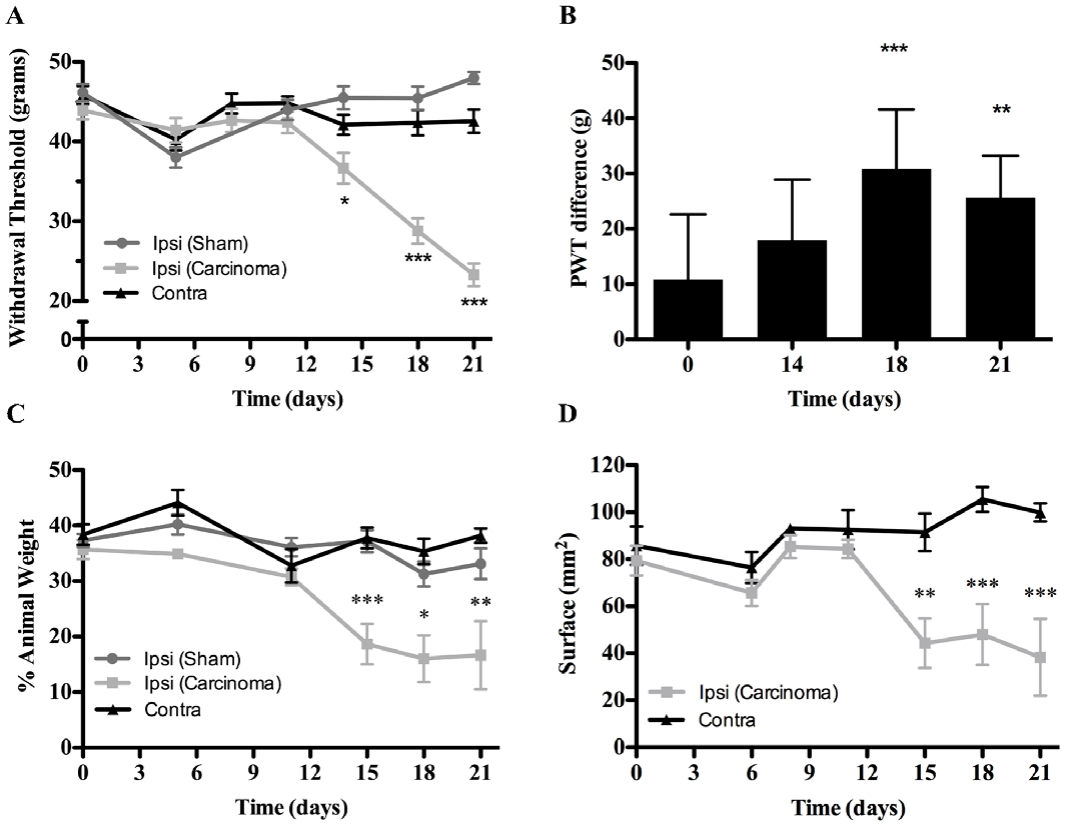
Evaluation of mechanical sustained pain on a 21-day schedule following the implantation of MRMT-1 cells in rat right distal femur metaphyse. (**A**) Allodynia was determined using the von Frey test following tumor implantation. The withdrawal threshold diminishes progressively from day 11 in response to nociception accommodation in cancer-bearing animals (n = 10). The contralateral paw remains unaffected, as does the sham-treated ipsilateral limb (n = 10). (**B**) Evaluation of hyperalgesia was performed using the Randall-Sellitto test. Results are expressed as the paw withdrawal threshold (PWT) difference between the ipsi- and contra-lateral hind paws. Significant hyperalgesia was observed at days 18 and 21 (n = 10). (**C**) Quantification of dynamic weight-bearing in the implanted animals, expressed as a percentage of animal weight. A significant difference in weight distribution on each paw is observable from day 15. (**D**) Dynamic analysis of the surface of contact between the hind paw and the sensor presents similar results at day 15, 18 and 21 when the ipsi- and contralateral paws are compared in cancer groups (n = 7 in each group). Data are mean ± S.E.M, *: p≤0.05; **: p≤0.01 ***: p≤0.001.

In the attempt to mimic clinical bone cancer pain, we performed a local injection of the cells directly in the femur between the medial epicondyle and the adductor tubercle. The impact of surgery was minimized by implantation at this anatomical site, which reduces the chances of patellar ligament or joint damage. Accordingly, as assessed by von Frey and dynamic weight bearing (DWB), sham animals showed no signs of mechanical allodynia or locomotion impairment throughout the behavioral test period (Figs. 1A, C), even in early days following the surgical procedure (data not shown). Cancer-bearing male rats inoculated with 30,000 MRMT-1 cells displayed a decreased withdrawal threshold in the dynamic von Frey test from day 14 (36.7±2.0 g; p<0.05; Fig. 1A). After that, allodynia rapidly progressed until day 21, when it was maximal (23.3±1.5 g, p<0.001). Cancer cells inoculated in the right hind paw did not elicit mechanical allodynia on the contralateral side (Fig. 1A). Concomitantly to allodynia, mechanical hyperalgesia developed progressively in tumor-bearing rats. As measured with the Randall-Selitto test, a significant difference was observed between ipsilateral and contralateral paw withdrawal thresholds from day 18 (30.9±10.8 g, p<0.001) to day 21 (25.7±7.6 g, p<0.01; Fig. 1B). These results demonstrate that measurable touch-evoked pain behaviors develop after the injection of carcinoma cells into the femur, as previously demonstrated with MRMT-1 cell implantation in the tibia [24]. After pain detection on day 14, subcutaneous administration of morphine (3 mg/kg) did not reverse mechanical allodynia induced by tumor progression in cancer-bearing rats (Figure S1).

Limb discomfort was first assessed by static weight-bearing. From day 15, half of the ipsilateral weight was redistributed to the contralateral paw (data not shown). In order to evaluate a representative weight redistribution across the animal's entire body, without restraint, we performed DWB assessments. The kinetics of transfer to the hind paws were similar to those observed in static bearing, starting around day 12 and increasing until day 21 (Fig. 1C). However, it is noteworthy that weight compensation was mainly made upon body parts other than the contralateral hind paw, such as the tail, bottom abdomen or forepaws. Indeed, at day 21, approximately 50% of the weight was borne elsewhere than the hind paws, in comparison to 33% prior to pain onset (data not shown). Accordingly, the paw surface in contact with the floor was also significantly affected from day 15, as compared to the contralateral paw (Fig. 1D). These pain behaviors mirror the clinical observation of breakthrough pain, which is experienced after the movement of tumor-bearing limbs in bone cancer patients.

### Neuronal and glial plasticity in cancer-bearing animals

**Figure 2:**
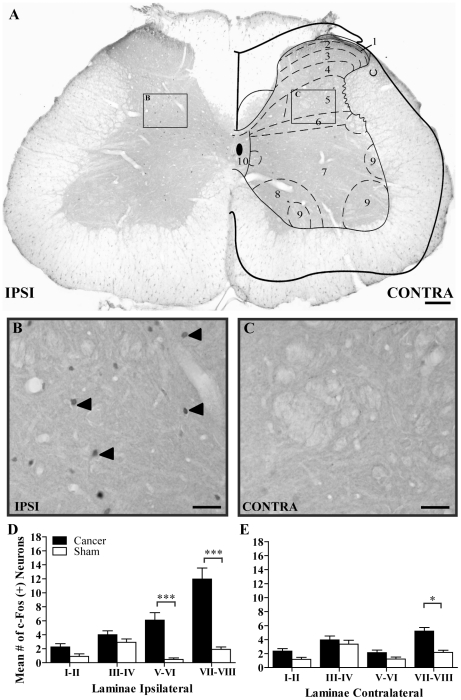
Expression of c-Fos protein in neurons of the spinal cord of bone cancer-bearing animals 14 days after implantation into the femur. (**A**) Photomicrograph of an L3 section of the spinal cord of a tumor-bearing rat depicting c-Fos protein's global expression profile. (**B–C**) High magnification of the ipsilateral and contralateral dorsal horn laminae V-VI, respectively. Black arrowheads point to c-Fos proteins only in the ipsilateral limb. (**D–E**) Histogram representations of Fos-LI neurons per laminae in cancer animals for ipsilateral and contralateral sides, respectively. Data are mean ± S.E.M. number of Fos-LI neurons in the different spinal cord segments for sham and cancer rats (Scale bars, A: 200 µm; B,C: 30 µm; n = 23 sections from 3 rats/group; *: p≤0.05; ***: p≤0.001).

**Figure 3:**
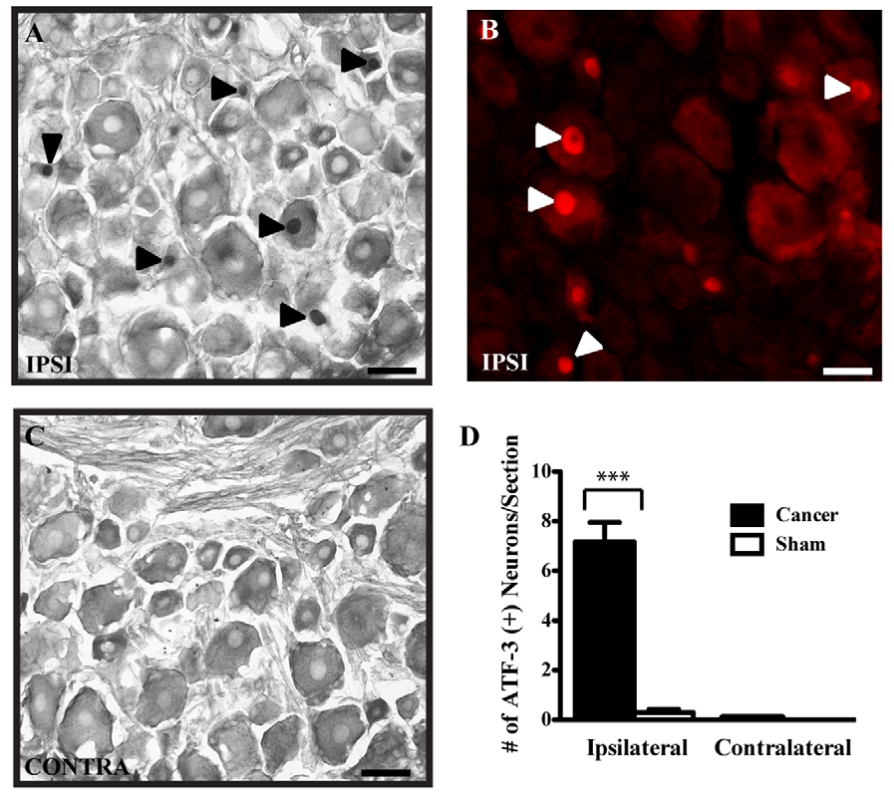
ATF-3 immunoreactivity in DRG neurons of bone cancer-bearing animals 14 days after implantation into the femur. (**A**) Primary ispilateral L3 DRG sub-populations of sensory neurons showing ATF-3-IR-positive neurons marked by dark labeled nuclei with DAB staining (black arrowheads). (**B**) ATF-3 immunofluorescence labeling was performed to confirm the presence of Fos reactivity (white arrowheads). (**C**) Immunohistochemistry for ATF3 in the corresponding contralateral DRG. (**D**) Histogram representation of ATF-3 positive neurons per section in cancer and sham animals. Data are mean ± S.E.M. number of ATF3-LI neurons (n = 24 sections from 3 rats/group; ***: p≤0.001; scale bar: 50 µm).

Metastasis proliferation in the rat femur induces neuronal activation and glial changes throughout afferent fibers projecting to L1-L3 spinal lumbar segments. Fourteen days following MRMT-1 cell implantation, immunohistochemical analyses were performed on spinal cord and DRG tissues to examine the resulting neurochemical alterations. C-Fos and ATF-3 immediate-early genes were used to follow spinal and sensory neuronal activities (Fig. 2 and 3, respectively). First, we observed a repetitive and scattered c-Fos expression pattern in the ipsilateral spinal horn of tumor-injected rats (Fig. 2A). At day 14 post-surgery, an intense increase in the number of c-Fos-immunoreactive neurons was observed towards deeper laminae V-VI and VII-VIII in spinal cord segments L1-L3, as compared to sham animals (Figs. 2B, D). In contrast, C-Fos staining was almost absent in the contralateral side (Figs 2C, E). As the tumor cells filled the intramedullary space of the bone, a significant up-regulation of ATF-3 expression also occurred in sensory neurons of the ipsilateral DRG (Figs 3A, B). At 14 days of cancer development, ATF-3 reactivity was absent in sham and contralateral sensory neurons (Fig. 3C). Typically, ipsilateral cancer DRG sections displayed seven times more ATF3-positive neurons than their corresponding contralateral part or the sham group (Fig. 3D). As previously observed in sarcoma-injected animals [11], substance P (SP) and isolectin B4 (IB4) immunoreactivities, labeling peptidergic and non-peptidergic small projecting nociceptive fibers, respectively, remained unchanged (data not shown).

**Figure 4:**
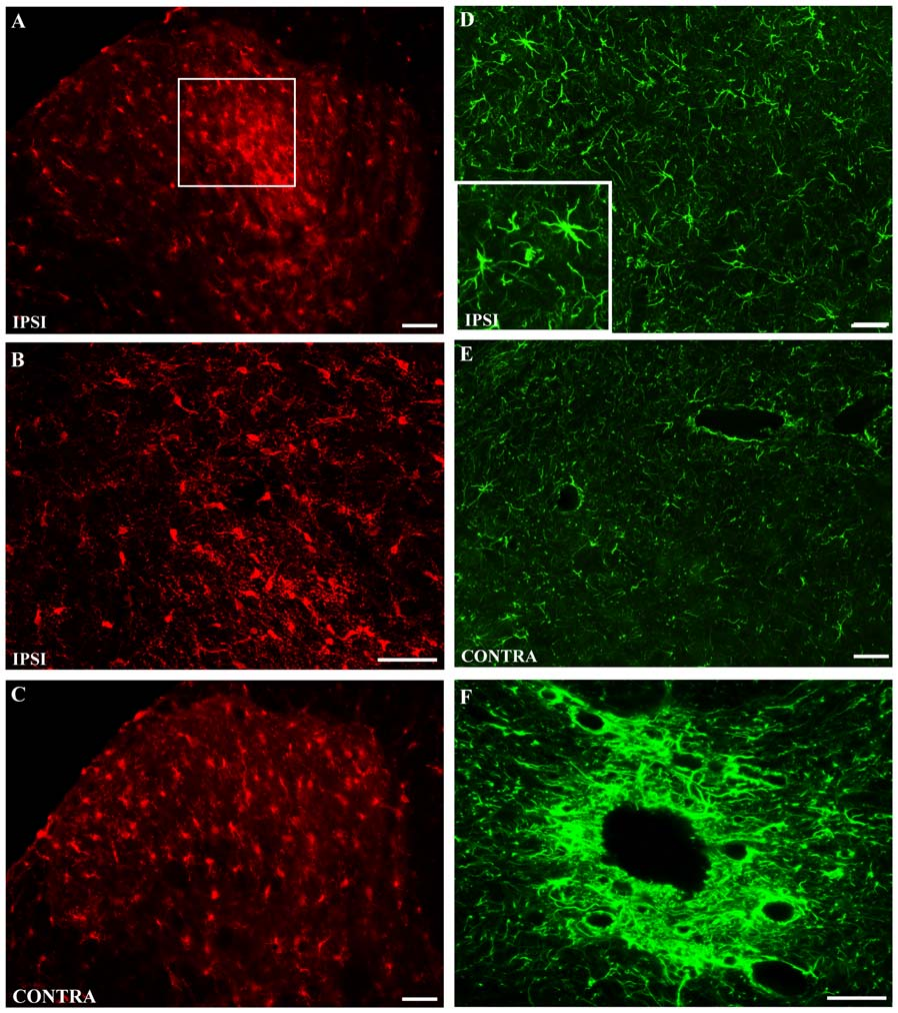
Microglia and GFAP staining in dorsal horn of the L3 spinal cord of rats at day 14 post-surgery. (**A**) An increase in Iba_1_ labeling is observable in the ipsilateral dorsal horn. (**B**) At high magnification (**insert**), glial cells are morphologically characterized by an increase in the length of distal processes. (**C**) The carcinoma does not induce alterations in the contralateral spinal cord microglial pattern. (**D**) The distribution is relatively similar in the ipsilateral superficial dorsal horn; however, astrocyte cell bodies are hypertrophied (**insert**). (**E**) On the contralateral side, astrocytes are distributed homogenously throughout the superficial laminae. (**E**) An increase in GFAP-IR is observed around the central canal in laminae X. Scale bars A,C,D,E: 50 µm; D insert: 15 µm; B: 30 µm; F: 100 µm).

Further, glial involvement was determined with Iba1 and GFAP markers, labeling microglia and astrocytes, respectively (Fig. 4). Microglia are recognized as pathology sensors capable of reacting to neuronal disturbances. Iba1 staining was increased in the ipsilateral dorsal horn 14 days after cancer cell inoculation of the femur (Figs. 4A, B). Iba1 fluorescence intensity was increased by 36% in the ipsilateral dorsal horn in comparison to its contralateral counterpart (p<0.05) and by 44% when compared to sham animals (p<0.05; Table 1). The labeling appeared to be widespread across dorsal horn laminae I-V, with the most prominent increase observed in laminae III-IV (Fig. 4A). Ipsilateral hot spots displayed, under higher magnification, dense clusters of amoeboid-shaped cells that are characteristic of activated microglia (Fig. 4B). Surrounding this intense gliosis zone, processes of microglial cells were rather ramified and branched outside laminae IV to VI, more representative of resting microglia, as observed on the contralateral side (Fig. 4C). There was no statistical difference in Iba1 labeling between sham animals and the contralateral side of cancer-bearing rats (Table 1). Normal and reactive astroglia were also detected 14 days post-surgery in L1-L3 spinal lumbar segments using GFAP (Fig. 4). Ipsilaterally, cancer-bearing rats displayed hypertrophic astrocytes characterized by cell body enlargement and extensive arborization of their distal processes (Fig. 4D and insert). In contrast, GFAP-labeled astrocytes appeared in their basal, inactivated morphological state in sham animals and on the contralateral side (Fig. 4E). This astrogliosis observed at the immunohistological level was, however, not reflected in the quantification of GFAP staining (Table 1). Interestingly, the central canal area (lamina X) was highly labeled in lumbar spinal cord segments that receive primary afferent input from the affected femoral bone (Fig. 4F). These results indicate that both neuronal and glial cells were activated ipsilaterally during bone cancer development.

**Table 1:** Quantification of glial activity at the lumbar level of spinal cord in bone cancer after 14 days.

	Iba1		GFAP	
	Ipsi	Contra	Ipsi	Contra
**Cancer**	19.04±4.96	12.24±2.18#	48.02±3.61	46.97±4.20
**Sham**	10.83±1.97*	10.27±0.60	45.81±2.62	41.36±2.35

Fluorescence immunohistochemistry was performed and quantified in dorsal spinal cord (L1-L3) of cancer-bearing and sham rats (n = 8). IBa1 immunolabeling of microglia showed a significant increase in fluorescence intensity between ipsilateral and contralateral sides (#; p<0.05) and between carcinoma-implanted animals and sham-group ipsilateral sides (*; p<0.05). GFAP immunostaining of astrocytes did not reveal any significant difference in fluorescence intensity thresholds between groups.

### Characterization of bone remodeling by faxitron and microcomputed tomodensitometry (µCT)

**Figure 5:**
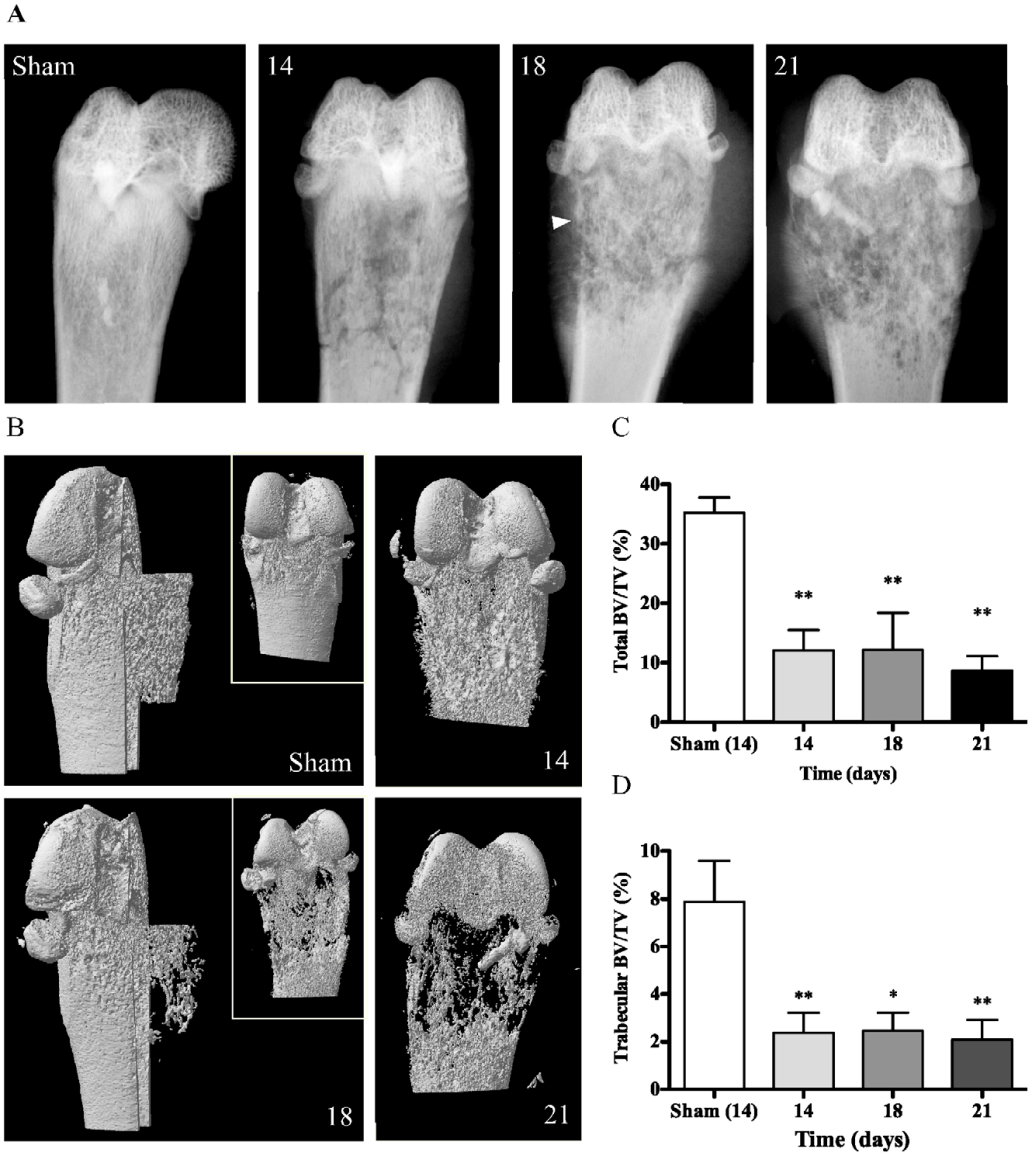
Radiological images of bone cancer-bearing rat femurs. (**A**) Faxitron anteroposterior radiographs of sham-operated and cancerous femur distal heads after 14, 18 and 21 days. At day 14, osteopenia is observed in the medullar canal of the bone while cortical integrity is maintained. At 18 days, the cortical line is blurred, characteristic of severe supracondylar osteopenia (arrowhead). Cortical bone integrity is partially compromised, contributing to the appearance of fracture. Twenty-one days after implantation, the bone shape is irregular due to anarchic matrix redeposition. An A3 metaphyseal complex fracture is present following such severe bone loss, affecting bone shape. (**B**) Microcomputed tomodensitometry of sham-operated and cancer-bearing rat femurs. The shaft represents cortical mineralized bone, while the extrusion represents trabecular bone. Sham femurs show intact structure and high cortical and trabecular densities. After 14 days, the metaphyseal cortical bone surface becomes irregular and porous, while the epiphysis remains intact above the growth plate. Trabecular reconstruction at day 18 reveals extensive bone loss, while the cortical architecture weakens. At day 21, the bone integrity is seriously compromised and marked by signs of potential fracture. (**C**) Bone modeling is quantified by the measurement of bone volume/tissue volume ratios, expressed in %. Throughout the entire bone structure, proportional bone content is significantly modified from day 14, with a greater decrease in bone percentage at day 21. (**D**) When the analysis is limited to the cancellous bone only, the same modifications, this time in trabecular architecture, are observed. (*: p≤0.05; **: p≤0.01; n = 4–5).

X-rays were taken to evaluate the impact of cancer proliferation on solid structures of the bone (Fig. 5A). The first acquisitions showed a progressively degrading metaphysis throughout 21 days of observation. Osteopenia gradually increased from day 14 and the cortical line was blurred from day 18 (white arrowhead). At day 21, a bulge shape was forming due to irregular deposition of bone matrix by osteoblasts. At that time, some animals presented a fragile structure characteristic of an A3 comminuted metaphyseal fracture. Micro X-ray computed tomography (µCT) was performed to provide insight into the loss in bone density and organization and to record trabecular bone morphometric parameters (modelization extrusion; Fig. 5B), as compared between sham and cancer-bearing rats. Scans showed a rough aspect from day 14 and severe progressive bone loss was observed at days 18 and 21. The dental amalgam was still in place and unaffected in the 21-day implanted femur. A global structural degradation was demonstrated by a decreasing volume ratio of whole bone structure (BV) over total volume of tissue (TV) in the region of interest (whole bone BV/TV; 65.8% and 75.3% decrease at days 14 and 21, p<0.01; Fig. 5C). More precisely, the level of trabecular remodeling, determined by the ratio of trabecular BV over TV, was significantly lower in cancer-bearing animals compared to shams at day 14 (74.8% decrease), 18 (93.9%) and 21 (88.4%) (p<0.05 and 0.01; Fig. 5D). By extrapolation, cortical bone degradation thus accounted for a large proportion of the degradation. Trabecular architecture weakening was also exposed by an increasing trend in trabecular pattern factor (+78.7% on day 18 (not shown)), representative of lower connectivity between bone fragments within the medullary canal. This altered architecture of the spongy bone may be attributable to tumoral and osteoclastic activity as well as to compensatory bone remodeling. The trabecular number, an indicator of bone density within the region of interest (ROI), was also drastically decreased at day 21 (80.1% of sham group, p<0.05; not shown). Finally, these results support the osteolytic character of the tumor in the femur of male rats as exposed on X-rays.

### Monitoring tumor proliferation and bone invasion by PET/MRI

**Figure 6:**
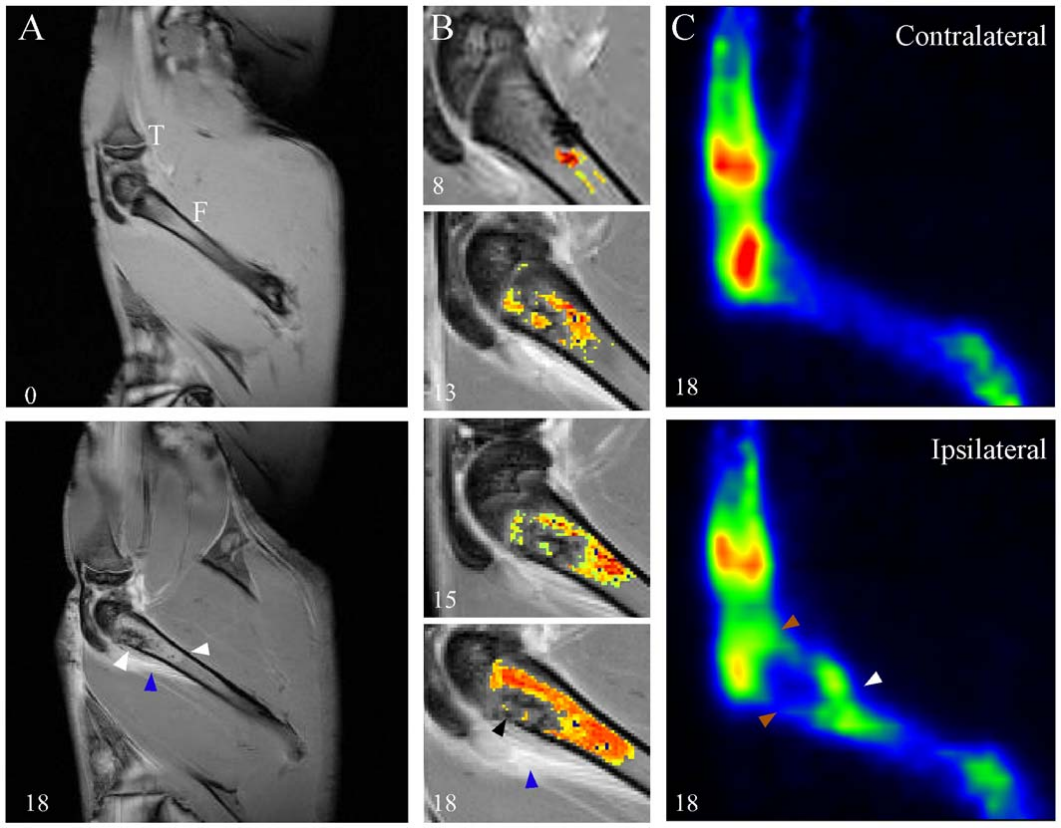
Medical imaging of bone tumor using MRI and PET. (**A**) Contrast-enhanced (Gd-DTPA) T1-weighted magnetic resonance (MR) images of a rat coping with femoral bone cancer. Bone images of the proximal tibia (T) and whole femur (F) show dispersion of the contrast agent in normal (**top**) and cancerous (Day 18; **bottom**) bones in a sagittal plane (n = 8). Cortical bone shows lower intensity (black), while soft-tissue areas are in shades of gray. Tumor proliferation affects the regular cortical line at day 18. Hyperintense areas within the medullary channel represent the tumor-affected distal part of the femur (white arrowheads). Enhanced areas outside the bone are characteristic of inflammation and edema (blue arrowhead). (**B**) Sagittal plane close-ups of the distal femur at different stages of tumor progression. Images are superimposed to artificial colors corresponding to voxel intensities and are indicative of tumor proliferation in the medullary channel. Tumor is detected at day 8 and its intensity and surface increase consistently throughout the experiment. Blue and black arrowheads label periosteal inflammation and necrotic clusters, respectively. (**C**) Sodium ^18^fluoride positron emission tomography scans of contralateral and ipsilateral cancer-bearing bones at day 18 post-cancer cell inoculation. The positioning remains the same between MRI and PET scans. Contralateral bone shows a high ^18^fluoride intake at the trabecular bone level, while the shaft of the femur remains lightly labeled. In contrast, the cancer-affected bone displays a markedly decreased signal at the distal extremity (orange arrowheads), while a high intensity signal is observable at the level corresponding to the proximal edge of the tumor area (white arrowhead). This is representative of substantial bone remodeling (n = 3).

MRI offers an excellent soft-tissue contrast [41] and information on tissue perfusion and microvessel permeability. Na^18^F PET provides an assessment of bone integrity and turnover. When combined, these two imaging modalities yield a more complete picture of pathological bone status. Sagittal gadolinium-enhanced *T*_1_-weighted MR images of the tumor-implanted rats were acquired at days 0, 6, 8, 10, 13, 15, 18 and 21 (Fig. 6A). Artificial coloring of a selected region of interest of the sagittal images using thresholds established from a signal intensity histogram highlights the time-dependent nature of tumor progression (Fig. 6B). The intact femur at day 0 presented a regular cortical line and even perfusion of the bone marrow by contrast agent Gd-DTPA. At day 18, diffuse hyperintense medullary infiltration was observed throughout the diaphysis, corresponding to the tumor's spread (white arrowheads). The femoral shaft acquired a rougher aspect, with a fading cortical line proximal to the metaphysis. The bulge shape of the femoral head was consistent with the X-ray observations (Fig. 5A). Contrast enhancement was also observed in the surroundings of the bone, indicative of possible periosteal inflammation and edema (Figs. 6A, B; blue arrowheads). The medullary cavity was gradually invaded and the presence of hypointense regions was emphasized, characteristic of necrotic islets and amalgam artifacts at the site of injection (Fig. 6B; day 18, black arrowhead). Notably, the tumor was detected as early as day 8 post-implantation, thus 6 days prior to pain onset, as measured in the dynamic von Frey test (Fig. 1A). Na^18^F PET images were co-registered to MR images for morphological reference (Fig. 6C). Fluoride ions are chemisorbed on bone hydroxyapatite crystals, providing insights on bone metabolism and structure and instantly reflecting increased uptake in the surroundings of bone metastases [42]. This procedure is highly specific, indicative of bone turnover and of tumor aggressiveness at the time of the scan, a methodology that would otherwise require multiple sessions of CT scans to detect a pathological evolution. Day 18 images displayed a photopenic lesion at the distal metaphysis in comparison to the contralateral side (Fig. 6C; orange arrowheads). Moreover, the ipsilateral border of the metaphysis/diaphysis region was marked by intense sclerotic bone activity (white arrowhead). The latter phenomenon is characteristic of a predominantly lytic pattern in a mixed bone tumor, as encountered in the clinic [43], [44].

### Histological overview of bone remodeling during tumor proliferation

**Figure 7:**
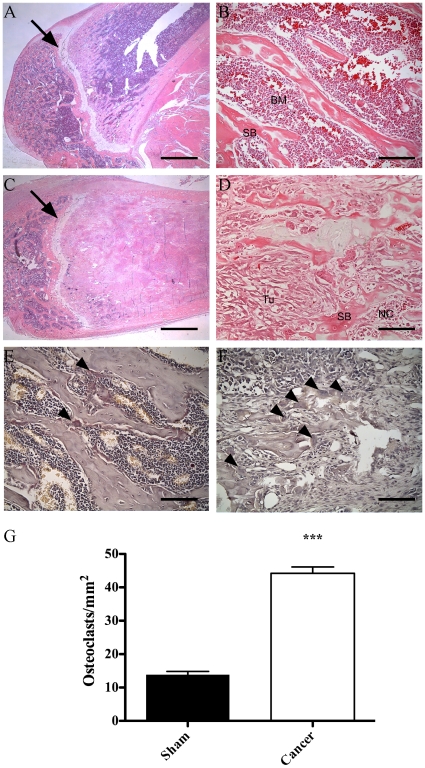
Hematoxylin & eosin coloration and quantification of TRAP staining in the rat distal femur at day 21 post-surgery. (**A–B**) Photomicrograph representative of the sham surgery group showing healthy bone structures (BM  =  Bone Marrow; SB  =  Spongy Bone). (**C–D**) Cancer-implanted bone illustrating an unstructured architecture. Note that the cartilage is still unaffected (arrows). Hematopoietic cells from the bone marrow are entirely replaced by proliferating MRMT-1 cells (Tu). Necrotic clusters (NC) appear in early areas of proliferation. Cancellous bone is sparsely deposited in a mosaic pattern, yielding fragile and disorganized bone. Larger lacunae and immature bone are indications of compensatory reconstruction. (**E–F**) Photomicrographs of trabecular bone stained with the TRAP coloration technique. Multinucleated active osteoclasts, bordering the hydroxyapatite matrix, are stained in brown-red (black arrows) in sham (**E**) and cancer-bearing (**F**) rats. (**G**) The number of differentiated osteoclasts per mm^2^ is three times greater in cancer rats than in sham animals, explaining the accelerated degradation of bone structure. ***: p≤0.001 (n = 12 for sham and n = 20 for the cancer group). Scale bars A,C: 1 mm; B,D,E,F: 90 µm.

The severity of the cancer model was also confirmed by classic histological approaches used to support non-invasive imaging data. The H&E staining enabled us to contrast healthy structures in the sham-operated group (Figs. 7A, B) after 21 days to cancer-bearing rats (Figs. 7C, D). Heavy signs of degradation, depicting an imbalance in osteo-clast/blast homeostasis, were identified in the trabecular spongy bone and ultimately replaced by necrotic clusters of tumor cells. Hematopoietic cells from the bone marrow were superseded by carcinoma cells. The cortical line was slender and irregular. However, we noted that despite the aggressiveness of the MRMT-1 cells, the calcified cartilage was unaffected and acted as a barrier to femoral epiphysis invasion (arrows). The chaotic architecture of the femur metaphysis structure in cancer-bearing animals was indicative of substantial lytic activity. We then evaluated the number of active multi-nucleated osteoclasts per surface, as measured using the Tartrate Resistant Acid Phosphatase staining protocol, which revealed brownish cytoplasmic staining indicative of osteoclasts (Figs. 7E, F, arrowheads). The number of osteoclasts per square millimeter was three times greater in the cancer group than in the sham-operated group (Fig 7G; p<0.001). This massive lytic activity accounted for the severe osteopenia observed, which increased the risk of fracture within 21 days of experimentation.

## Discussion

Bone cancer pain is a complex clinical syndrome; the mechanism remains to be elucidated. Advanced breast cancer often evolves towards bone dissemination and severe pain. Pre-clinical animal pain models mimicking relevant clinical observations will help to elucidate the mechanisms underlying the generation of pain and facilitate the development of preventative and palliative therapies.

### A new model of bone cancer pain

Based on clinical observations, the femur is the most frequent site of breast metastases in long bones [28]. It is also the second most common site (35% of cases) of pathological fracture induced by bone cancer [45]. To this day, disseminating breast-to-bone models, although relevant, lead to high variability in the targeted sites, rendering study of the mechanisms of bone cancer pain genesis and maintenance difficult to isolate [32], [33], [46]. On the other hand, models using local bone implantation to mimic cancer pain often use cell lines from primary cancers such as fibrosarcoma [15], [30], [47], [48], [49], [50]. Bone metastases secondary to breast cancers are more likely to induce chronic pain. Therefore, in the present study, we developed a model of bone cancer pain induced by the femoral invasion of breast carcinoma in the male rat. We cautiously chose to use male rats to avoid hormonal variations that are well-known to influence pain response [51], [52] and considering the fact that we demonstrated that MRMT-1 cells express estrogen receptors. We felt that it was preferable to first pursue our investigation on males in order to limit those variations and compare the evolution of pain and the progression of bone remodeling to other reported cancer pain models. Indeed, MRMT-1 cells have been abundantly used in the past to assess bone cancer pain following breast cancer cell inoculation in either males [53], [54], [55], [56], [57] or females [9], [20], [21], [24], [58], [59]. The behavioral, immunohistochemical, pathological and medical imaging approaches used to characterize, provide evidence that suggests a resemblance to the chronic and invasive pathology that develops in humans [60]. Importantly, we opted to minimize impairment due to the implantation step. Thus, the tumor is the exclusive source of pain and we avoid confounding variables (e.g., early inflammation, transient pain, locomotion discomfort attributable to surgery). Recent models of bone cancer pain, targeting the femurs in mice, consisted in arthrotomia, displacement of the patellar ligament and knee joint alteration [24], [30]. Affecting the subchondral bone can expose nociceptors in regions susceptible to biomechanical forces, thus amplifying weight-bearing pain [61]. The implantation approach suggested here does not result in pain or extraosseous inflammation.

### A relevant evaluation of behavioral pain

Breakthrough pain episodes frequently occur as the tumor infiltrates the bone matrix. These transitory episodes of extreme pain are evoked by non-noxious movement or mechanical loading of the tumor-affected bone. Breakthrough pain at rest (spontaneous) and during ambulation (volitional) is the first presenting characteristic of patients consulting a physician for metastatic disease, is highly prevalent in bone cancer patient cohorts, and is also an indicator of fracture risk [62], [63], [64], [65]. However, stimulus-evoked withdrawal thresholds remain the mainstay of pain evaluation. While we evaluated the consistency of the development of touch-evoked pain in previous bone cancer studies [13], [24], we investigated volitional pain to maintain clinical relevance. Static weight bearing has been used to determine the difference between cancer-bearing and healthy paws. However, the animal is restrained and stressed, masking information that could potentially be used to describe volitional pain. Consequently, we tested a dynamic weight-bearing system to monitor weight distribution during free ambulation in an open enclosure. The weight borne by any standpoint of the animal and the surface of contact were measured over an extended period of observation by floor captors. Therefore, we gained a greater appreciation of volitional pain, supported by the fact that our animals showed no discomfort following the implantation procedure. Additionally, we demonstrated that weight was redistributed entirely upon standpoints other than the contralateral hind paw, such as the tail and front paws. Thus, analgesics proven efficient in reversing weight-bearing to initial values in a static apparatus might not reflect reality. To avoid bias in evaluation of analgesic potency, quadrupeds should be tested in a natural ambulation position, just as humans would be tested in a standing posture. Therefore the model proposed here should be more persuasive in the attempt to evaluate the effect of analgesic treatments on volitional pain, ultimately resulting in more successful transfer of animal models to the clinic [64].

#### Neurochemical changes occurring in bone cancer pain

Bone cancer generates a profound reorganization in the DRG and spinal cord at both neuronal and glial levels. As previously observed in rat sensory and motor neurons following spinal and sciatic nerve injury [66], [67] and recently in mouse bone cancer models [15], [68], [69], [70], ATF-3 was shown to be up-regulated in DRG neuron somas 14 days after tumor implantation. This neuronal hyperactivity accompanying hyperalgesia and allodynia is also detected in the spinal cord, reflecting the central sensitization phenomena frequently observed in chronic pain states. In the present model, c-Fos-expressing neurons were significantly increased in deeper laminae V-VIII of the spinal cord following MRMT-1 carcinoma cell inoculation. However, in a previous study, sarcoma-induced c-Fos activation was observed in superficial layers I-II of the dorsal spinal horn [30], [71]. In neuropathic pain, non-nociceptive Aβ fibers are stimulated, increasing neuronal firing towards lamina V-VI of the spinal cord [72]. Therefore, Aβ fiber over-activation may be related to acidic tumor environment or nerve compression, thus contributing to c-Fos activation in deep layers. In human, the quality of bone cancer pain is marked by substantial heterogeneity, either due to the cancer or resulting from the alteration of distinct bone structures. These discrepancies in c-Fos expression may therefore reflect sensory inputs conducted by unmyelinated or myelinated fibers innervating either the bone marrow, the mineralized bone, or the periosteum.

Glial cell phenotypic changes were also marked by astrocyte hypertrophy and distal processes of increased length, in both the dorsal and ventral ipsilateral lumbar L1-L3 spinal cord. Recruitment of activated astrocytes may contribute to the initiation and maintenance of persistent cancer and neuropathic pain states, as previously reported [73], [74]. Interestingly, GFAP staining was intense in lamina X, a region known to relay visceral information. Tumor invasion of the marrow and the sinusoid walls of the distal metaphysis is likely to sensitize nerve fibers at this site and stimulate central astrocyte recruitment. Using a neurotropic viral approach, Denes and colleagues [75] infected bone marrow and demonstrated retrograde neuronal labeling of the central gray matter in lamina X of lumbar segments after 4 days. This is in line with a high prevalence of referred visceral pain in the cancer population. In the present model, we also found a substantial increase in microglia in the ipsilateral dorsal horn, concurrent to the observations made by Zhang and colleagues [27] in the rat but in opposition to the unchanged staining previously obtained with OX-42 in mice [30], [71]. Microglial activation was shown to peak after 1 week of injury and to decline over several weeks thereafter [76]. However, most of the bone cancer research groups performed analyses of duration ranging from 19 to 21 days. In our case, at day 14, we were able to observe hypertrophied microglial bodies in laminae V and a large number in other laminae, factors that represent a post-active or latent state in response to an insult. Whether microglia reactivity appears earlier than the activation of astrocytes in this bone cancer-induced pain model awaits further investigation.

### Detection of early bone metastases by a clinical imaging approach

Most of the clinical problems associated with metastatic bone diseases are a result of tumor-induced dysregulation of abnormal bone turnover. The development of non-invasive imaging modalities (PET/MRI) providing early diagnosis of bone remodeling and allowing physicians to monitor the therapeutic response has provided advantages beyond those of plain film radiographs. Recent human studies also showed that Na^18^F PET is more sensitive than ^99^Tc radionuclide bone scan in detecting metastases. In fact, Na^18^F PET allows physicians to detect all osteolytic metastases, while radionucleide bone scan detects only 49.3% of metastases [77]. Furthermore, a recent study revealed that MRI is superior to CT in detecting the presence and extent of bone marrow metastases [78]. CT could only identify 17% of these tumors, while MRI could reveal the presence of 83%. This is of crucial importance when choosing the appropriate treatment, as 24% of the patients saw their treatment modified after an MRI, as compared to the treatment prescribed after a prior CT scan [78]. Accordingly, PET/MRI holds the best potential to assess the potency or efficacy of tumor-suppressing treatments at preclinical and clinical levels [79].

Bone resorption and tumor growth are the most important aspects of pain onset [8], [68]. Therapeutic validity would therefore be supported by an appropriate correlation between tumor development and activity profiles versus pain intensity. Our results reveal timely and reproducible detection and monitoring of bone tumor growth in the same animal by MR imaging. This proves useful, considering that clinical and preclinical PET/CT analyses are limited by the amount of ionizing radiation applied to the subject, which can potentially reduce the frequency of follow-up sessions. Advantageously, MRI techniques developed for small animals are often adapted for use in humans. Further, our MRI studies allowed us to detect the tumor at day 8 post MRMT-1 cell inoculation, prior to pain onset in every case. Thus, the mechanical compression of bone tissues and the algogenic substances released by the bone environment and tumor at day 8 are not sufficient to exacerbate nociceptors and stimulate abnormal neuronal firing. Thus, detecting bone metastases before symptomatic pain would support a prophylactic approach in metastatic disease management. PET images showed decreased ^18^F accumulation in the area affected by the tumor after administration of Na^18^F. This confirms the high lytic character of the mammary carcinoma, since the high affinity of ^18^F for bone hydroxyapatite normally results in large signal intensities in the normal trabecular bone. In contrast, accumulation of ^18^F in the region surrounding the tumor was observed, indicative of active bone turnover. Increased osteoblastic activity induced in an attempt to compensate for the lytic activity of the tumor could account for the enhanced signal observed [7]. Indeed, a high number of osteoclasts were detected in the regions adjacent to the tumor in histological sections, and anarchic bone turnover was also seen in the femur metaphysis in cancer-bearing animals. Altogether, these observations show that metastases-induced loss of integrity in the tumor-bearing bone contributes to promoting movement-evoked breakthrough pain in cancer patients. Indeed, prevention of bone metabolism imbalance, through bisphosphonates therapy for example [18], in combination with early treatment of tumor progression and pain development – as well as their timely evolution – could be monitored using PET and MRI co-registration technology.

### Aiming for osteoclastogenesis

Breast cancer usually displays a dominant lytic profile when it metastasizes to the bone [80]. This fact is also observed in our cancer model, as determined by µCT analyses that reveal a striking decrease in trabecular bone content. This is in accordance with the dense and slurry vascularization present within the trabeculae, rendering the bone ideal for embolus nesting. Tumor expanded beyond the limit of the compact cortical bone, demonstrating the aggressiveness of the MRMT-1 cells in infiltrating and degrading the bone matrix. Extra-femoral spicules, characteristic of compensative bone formation were detected, consistent with other studies [7]. The trabecular pattern, an indicator of connectivity within the trabecular architecture, was strongly increased in cancer-bearing bones. In humans, biopsies are used for trabecular architecture evaluation [81], [82]. Clinically, a higher trabecular pattern factor is correlated with a higher risk of fracture [83]. Close monitoring of these parameters and manifestations is important to minimize pain.

Several observations from the present study emphasize the crucial role of osteoclasts in cancer-induced bone loss and in the etiology of bone cancer pain. Histological analyses demonstrated that tumor progression had a dramatic effect on osteoclast activity. Indeed, TRAP-positive multinucleated cells tripled in number and displayed typical hypertrophic bodies. This explains, in part, the decreased bone volume observed using the µCT and the decreased ^18^F uptake in the most affected areas of the bone. The tumor releases osteoclast maturation factors or stimulates osteoblast secretion of RANKL and down-regulation of osteoprotegerin, resulting in dominant resorptive activity [9], [84], [85]. In addition to prostaglandins and other factors released by the tumor, bone resorption results in the release of protons that will stimulate nociceptors. The number of osteoclasts is therefore an indicator of potential bone pain onset and should be screened in every bone cancer model.

### Conclusion

While pain occurs at any time point during the course of the disease, in general, the more advanced the cancer, the more likely it is that the patient will experience intractable pain. The development of treatments to reduce the incidence of bone metastases is therefore of great clinical significance to improve the quality of life of bone cancer-bearing patients. Medical imaging at the site of the tumor in combination with pain assessment is the key to determine the efficacy of cancer treatments. Indeed, the evaluation of tumor progression could elucidate the onset of pain. With recent reports on the adverse effects of analgesic therapies on bone homeostasis [15], skeletal structure must be monitored during follow-up, in parallel with chronic pain management. The methods we propose in this animal model are based on clinical multimodal approaches and are intended to optimize the research and development of therapeutic strategies for bone cancer pain.

## Supporting Information

Figure S1 Evaluation of the effect of subcutaneous morphine on bone cancer pain induced by MRMT-1 breast carcinoma inoculation. Mechanical allodynia was evaluated following acute administration of 3 mg/kg of morphine sulfate (MS), starting at day 6. The paw withdrawal threshold (PWT) was evaluated 30 minutes after morphine injection. After pain detection on day 14, repeated morphine treatments did not induce any significant analgesic effect in cancer-bearing animals as compared to non-treated tumor-bearing rats. Note that subcutaneous administration of morphine was antinociceptive at day 6, just before cancer-induced pain (** p<0.01; n = 7).
(0.06 MB TIF)
