# Supplementary figures and images for: Behavioral, Medical Imaging and Histopathological Features of a New Rat Model of Bone Cancer Pain
Source: PLoS One. 2010 Oct 29;5(10):e13774. doi: 10.1371/journal.pone.0013774 (PMC2966439; doi:10.1371/journal.pone.0013774)

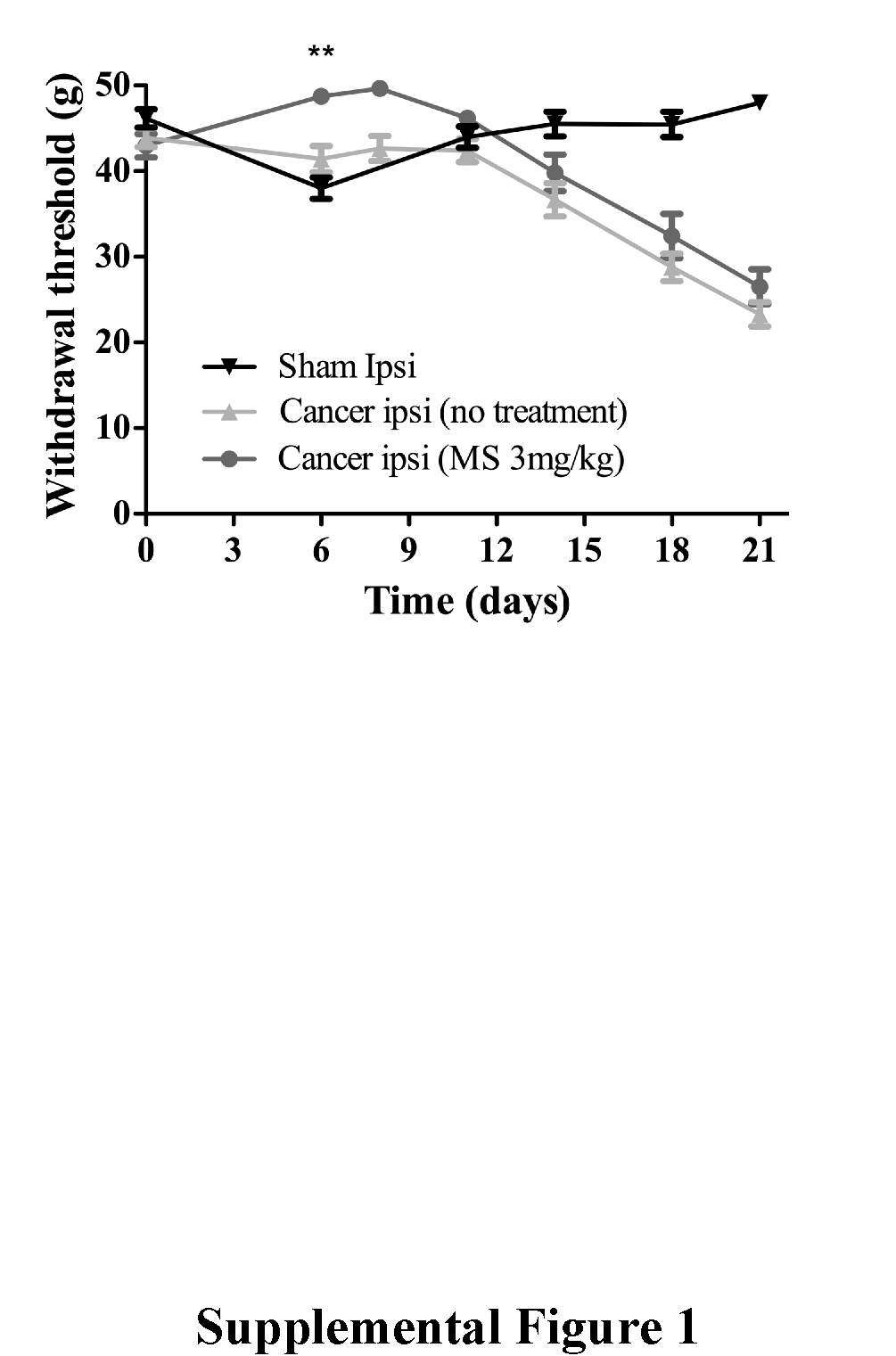

Supplement: Figure S1 — Evaluation of the effect of subcutaneous morphine on bone cancer pain induced by MRMT-1 breast carcinoma inoculation. Mechanical allodynia was evaluated following acute administration of 3 mg/kg of morphine sulfate (MS), starting at day 6. The paw withdrawal threshold (PWT) was evaluated 30 minutes after morphine injection. After pain detection on day 14, repeated morphine treatments did not induce any significant analgesic effect in cancer-bearing animals as compared to non-treated tumor-bearing rats. Note that subcutaneous administration of morphine was antinociceptive at day 6, just before cancer-induced pain (** p<0.01; n = 7). (0.06 MB TIF) [file pone.0013774.s001.tif]
